# Functional Significance of CD57 Expression on Human NK Cells and Relevance to Disease

**DOI:** 10.3389/fimmu.2013.00422

**Published:** 2013-12-09

**Authors:** Carolyn M. Nielsen, Matthew J. White, Martin R. Goodier, Eleanor M. Riley

**Affiliations:** ^1^Department of Immunology and Infection, London School of Hygiene and Tropical Medicine, London, UK

**Keywords:** CD57, NK cells, HCMV infection, ageing, chronic infection, cancer, autoimmune diseases, T cells

## Abstract

Historically, human NK cells have been identified as CD3^−^CD56^+^CD16^±^ lymphocytes. More recently it has been established that CD57 expression defines functionally discrete sub-populations of NK cells. On T cells, CD57 expression has been regarded as a marker of terminal differentiation and (perhaps wrongly) of anergy and senescence. Similarly, CD57 expression seems to identify the final stages of peripheral NK cell maturation; its expression increases with age and is associated with chronic infections, particularly human cytomegalovirus infection. However, CD57^+^ NK cells are highly cytotoxic and their presence seems to be beneficial in a number of non-communicable diseases. The purpose of this article is to review our current understanding of CD57 expression as a marker of NK cell function and disease prognosis, as well as to outline areas for further research.

## CD57 is a Marker of NK Cell Differentiation

CD57 was first identified on cells with natural killer activity using the mouse monoclonal antibodies Human Natural Killer-1 (HNK-1) (1) and Leu-7 (2) and was subsequently assigned the cluster of differentiation (CD) designation, CD57, at the fourth International Workshop of Human Leukocyte Antigens in 1989. HNK-1/Leu-7/CD57 was initially believed to be uniquely expressed on NK cells – and was used to define this population (1, 3) – although it was soon apparent that CD57 was expressed only on a subset of functionally distinct NK cells (4). CD57 was subsequently identified on CD8^+^ T cells (5–7) as well as cells of neural crest origin (1, 8–13). Indeed, it was the neuroscience community that ultimately defined CD57 as a terminally sulfated carbohydrate epitope (glucuronic acid 3-sulfate) (14–16). In neural cells, the CD57 epitope is predominantly restricted to adhesion molecules (17) but little attention has been paid to the precise identity of the molecules expressing the CD57 epitope on NK cells and T cells, precluding a full understanding of the relationship between CD57 expression and lymphocyte function. Although one study identified the CD57 epitope on the IL-6 receptor gp130 of resting lymphocytes (18), the cells expressing CD57/gp130 were not identified and no comprehensive analysis of CD57-expressing molecules on T cells or NK cells has been reported.

While first characterized as an NK cell marker, CD57 has been most widely explored as a marker of replicative senescence on T cells (19). Under conditions of persistent immune stimulation, memory T cells convert from CD28^+^CD57^−^ to CD28^−^CD57^+^ (20); CD57^+^ cells have short telomeres, low telomerase activity, low expression of cell-cycle associated genes and limited proliferative capacity (20, 21). However, CD57^+^CD28^−^CD8^+^ T cells can proliferate given an appropriate cytokine milieu (22), their sensitivity to apoptosis is disputed (23, 24), they are highly cytotoxic (25, 26) and express natural killer receptors (27). CD57^+^CD8^+^ T cells should thus be regarded as terminally differentiated, oligoclonal populations of cytotoxic cells generated in response to chronic antigen stimulation.

In light of the T cell data it was suggested that CD57 may also be a marker of NK cells with poor proliferative capacity and, perhaps, a degree of immunosenescence (21, 23, 28). Indeed, acquisition of CD57 on NK cells – following stimulation with IL-2 or coculture with target cells – correlates with maturation of the CD56^dim^ NK cell subset, with lower expression of NKp46, NKp30, NKG2D, and NKG2A, and higher expression of CD16, LIR-1, and killer cell immunoglobulin-like receptors (KIRs) (29). Similarly, in hematopoietic stem cell transplant recipients exposed to human cytomegalovirus (HCMV) infection, differentiation of CD56^dim^ NK cells involves acquisition of CD57, loss of NKG2A, gain of KIRs, and changing expression of homing molecules (30). These studies, together with experiments in Rag2^−/−^ γcR^−/−^ mice reconstituted with human hematopoietic stem cells and treated with IL-15 (30), and the observation that fetal and newborn NK cells lack CD57 (31), indicate that CD57^+^ NK cells differentiate from CD56^dim^CD57^−^ NK cells in an irreversible process with highly stable expression of CD57 likely being the final step in maturation (30, 32). This differentiation is accompanied by functional changes (29, 30): compared with CD57^−^ cells, CD57^+^ NK cells proliferate less well in response to IL-2 and IL-15 and produce less IFN-γ in response to IL-12 and IL-18, consistent with their lower levels of IL-12Rβ mRNA (29) and reduced surface expression of IL-2Rβ and IL-18Rα (30). On the other hand, CD57^+^ NK cells retain their cytolytic potential (30) and a proportion of CD57^+^ NK cells are able to produce IFN-γ after crosslinking of CD16 [Ref. (29); White et al. submitted] indicating that CD57^+^ NK cells are intrinsically able to produce IFN-γ but that they may have different activation requirements.

In summary, therefore, progression from CD56^bright^ to CD56^dim^CD57^−^ to CD56^dim^CD57^+^ reflects a maturation pathway for NK cells (33, 34) and rather than being a marker of anergy or immunosenescence, acquisition of CD57 represents a shift toward a higher cytotoxic capacity, greater responsiveness to signaling via CD16 and natural cytotoxicity receptors (NCRs) and decreased responsiveness to cytokines (29, 35). The extent to which CD57 expression *per se* drives these changes in function, as opposed to being a marker for cells with altered expression of other attributes of a mature NK cell, is not entirely clear and may represent a fertile area for further research. In addition, a much better characterization is required of the cell surface molecules that express the CD57 epitope, the mechanisms by which CD57 is induced on them, and its functional consequences.

## CD57 Expression and Cancer

Both CD8^+^ T cells and NK cells are able to kill tumor cells through mechanisms including perforin/granzyme-mediated cytolysis and TRAIL- or FAS-mediated apoptosis (36). Accumulation of CD57^+^CD8^+^ T cells is seen frequently in individuals with various forms of cancer (37) and has been associated with reduced survival in those with renal cell carcinoma (38), melanoma (39), gastric carcinoma (40), multiple myeloma (41), lymphomas, acute and chronic myeloid, and lymphocytic leukemias (42), among many other examples. CD57 expression on CD4^+^ T cells has also been associated with Hodgkin’s lymphoma (43) and chronic lymphocytic leukemia (44). This association between malignancy and expanded populations of CD57^+^ T cells is likely explained by persistent stimulation of these cells by tumor-associated antigens in the absence of effective tumor clearance (45).

NK cells were initially identified by their ability to kill malignant cells (46–48) and a large body of clinical and experimental evidence now supports their crucial role in cancer immunosurveillance (49). Reduced MHC Class I expression (50) and *de novo* expression of stress related molecules (such as B7-H6, MICA, MICB, RAE-1, MULT1, and members of the ULBP family) in malignant cells alter the balance of inhibitory (via KIRs and NKG2-CD94 heterodimers) and activating (via NCRs and NKG2D homodimers) signals for NK cells (51), leading to their activation. High frequencies of peripheral or tumor-associated CD57^+^ NK cells are reported in cancer patients and – in sharp contrast to what has been seen for CD8^+^ T cells – have frequently been linked to less severe disease and better outcomes (Table 1). This would be consistent with enhanced tumor surveillance/cytotoxicity of the mature, CD57^+^ NK cell subset (29); whether these associations are confounded by HCMV infection status (see below) is currently unclear. In the case of advanced gastrointestinal stromal tumors treated with the chemotherapeutic agent imatinib mesylate, NK cell secretion of IFN-γ after IL-12/IL-2 stimulation was correlated with improved long-term survival (52). Since CD57^−^ NK cells are the major subset producing IFN-γ in response to cytokines, this suggests that a heterogeneous NK cell population comprising both CD57^−^ and CD57^+^ subsets may be optimal for combating neoplasia. Clearly further studies, ideally longitudinal in nature and accompanied by data on potentially confounding factors, are needed to determine the roles of different NK cell subsets in combating different types of malignancies.

**Table 1:** **Associations between cancer prognosis and CD57 expression by NK cells**.

Cancer type	Observations	Reference
Acute lymphoblastic leukemia	Increased NK cell activity and increased numbers of CD57^+^ and CD16^+^ NK cells in bone marrow associated with complete remission	Sorskaar et al. (57)
Hodgkin’s disease	Absence/low number of CD57^+^ NK cells in tumor tissue (by immunohistochemistry) associated with relapse	Ortaç et al. (58)
Non-Hodgkin’s lymphoma	Higher numbers of intratumoral CD57^+^ NK cells are associated with relapse free survival in pediatric cases	Ortaç et al. (58)
Metastatic tumors in the brain	CD57^+^ NK cells infiltrate brain metastases of various origins (lung, breast, and renal carcinomas; melanoma) but no correlation between numbers of infiltrating CD57^+^ NK cells and apoptosis of malignant cells	Vaquero et al. (59)
Colorectal cancer	Increased CD57^+^ NK cells in germinal centers of draining lymph nodes, but rarely in primary or metastatic lesions; CD57^+^ NK cells may prevent establishment of tumor in lymph nodes?	Adachi et al. (60)
Bladder carcinoma	Lower frequency of CD56^+^ and CD57^+^ PBMC in patients with invasive and non-invasive tumors is correlated with reduced cytotoxicity against T24 bladder cancer cell line	Hermann et al. (61)
Breast carcinoma	Survival is positively correlated with the number of tumor infiltrating CD57^+^ NK cells and with expression of CX3CL1 (a known NK cell chemoattractant) by the tumor cells	Park et al. (62)
Gastric carcinoma	CD57^+^ NK cell infiltration associated with a lower clinical grade tumor, reduced venous invasion, fewer lymph node metastases, less lymphocytic invasion, and increased 5 year survival outcome	Ishigami et al. (63)
Oral squamous cell carcinoma	Low density of tumor infiltrating CD57^+^ NK cells and high numbers of TNF^+^ cells associated with higher clinical staging	Turkseven and Oygur (64)
Esophageal squamous cell carcinoma	Tumor infiltrating CD57^+^ NK cells positively associated with increased survival over 80 months	Lv et al. (87)
Squamous cell lung carcinoma	Tumor infiltrating CD57^+^ NK cells positively correlated with increased survival 2 years after surgery	Villegas et al. (88)
Pulmonary adenocarcinoma	Higher absolute numbers of tumor infiltrating CD57^+^ NK cells correlated with tumor regression	Takanami et al. (89)
Various	Low numbers of CD57^+^ NK cells in peripheral blood are associated with carcinomas of colon, lung, breast, and neck; no association was with melanoma or sarcoma	Balch et al. (90)

## CD57 Expression and Autoimmunity

Autoimmune diseases tend to be highly antigen-specific and mediated by autoantibodies or autoreactive T cells. In general, expanded populations of autoreactive CD57^+^ T cells are associated with more severe disease – Wegener’s granulomatosis (65), pars planitis (25), multiple sclerosis (MS) (66), type I diabetes mellitus (67), Graves’ disease (68), and rheumatoid arthritis (RA) (69), amongst others. This likely reflects killing of vital host cells by these highly cytotoxic lymphocytes (68), although the loss of T cells with immunosuppressive potential may also play a role (67).

Perhaps surprisingly, autoimmune disease is consistently associated with reduced frequencies or absolute numbers of circulating CD57^+^ NK cells and/or impaired NK cell cytotoxicity (Table 2) (70–78), suggesting that cytotoxic CD57^+^ NK cells may play a regulatory role, preventing or suppressing autoimmune disease. In MS, peripheral NK cells lose expression of FAS during relapse and regain it during remission (70) and FAS^+^ NK cells can inhibit myelin basic protein-specific T cell IFN-γ responses (79), suggesting that NK cells may regulate autoreactive T cells. On the other hand, chronic NK cell lymphocytosis (which is associated with peripheral neuropathy, arthritis, and vasculitis) is characterized by increased absolute numbers of circulating immature NK cells with low cytotoxicity (80, 81). Similarly, NK cells have been found in the inflammatory infiltrates of psoriatic skin lesions (82), in synovial fluid of joints affected by RA (83), and in pancreatic islets of type I diabetes patients (84). NK cells in the synovial fluid of patients with RA, and those infiltrating psoriatic skin lesions, are immature CD56^bright^ or CD57^−^ and able to secrete IFN-γ and TNF (85, 86), suggesting that they may contribute to the inflammation rather than suppress it (84).

**Table 2:** **Associations between autoimmune diseases or infections and CD57 expression by NK cells**.

	Observations	Reference
**AUTOIMMUNE DISEASE**		
Alopecia areata	CD57^+^ NK cells are significantly reduced in peripheral blood of patients with multiple foci of alopecia	Imai et al. (91)
Atopic dermatitis	Reduced frequencies of CD57^+^ NK cells in peripheral blood of patients compared to healthy controls, with greatest reduction in the most severe cases	Wehrmann et al. (126) and Matsumura (127)
Sjögren’s syndrome	Decreased numbers of CD57^+^ NK cells observed in peripheral blood of patients compared to controls	Struyf et al. (128)
IgA nephropathy	Decreased proportion of CD57^+^ CD16^+^ lymphocytes in the peripheral blood of patients compared to healthy controls	Antonaci et al.(129)
Psoriasis	NK cells infiltrating skin lesions – but also unaffected skin – are predominantly CD57low	Batista et al. (85)
**INFECTION**		
HCMV	Increased proportions of CD57^+^ NK cells in infected individuals; CD57 expression limited to the NKG2C^+^ subset	Gratama et al. (110), Lopez-Vergès et al. (99) and Foley et al. (111, 112)
HIV	In chronic infections, there is a loss of CD57-/dim NK cells, but the absolute number of CD57^+^ NK cells remains constant	Hong et al. (100)
Chikungunya virus	Increased proportions of CD57^+^ NK cells after infection in HCMV^+^ patients	Petitdemange et al. (115)
Hantavirus	NKG2C^+^ NK cell subset expanded during infection in HCMV^+^ patients and the majority of these cells are CD57^+^	Björkström et al. (114)
Hepatitis B and Hepatitis C	NKG2C^+^ NK cell population is expanded in chronic infections, and these are predominantly CD57^+^, but co-infection with HCMV appears to be the driver of this effect	Béziat et al. (97)
Lyme disease	Conflicting evidence on whether chronic disease leads to a reduced proportion of CD57^+^ NK cells in peripheral blood	Stricker et al. (117), Stricker and Winger (118), and Marques et al. (119)

Taken together, these data are consistent with the hypothesis that immature CD57^−^ NK cells may contribute to autoimmune inflammation and tissue damage whereas more highly differentiated, cytotoxic, CD57^+^ NK cells may fulfill an immunoregulatory role, possibly deleting chronically activated T cells, as in viral hepatitis (103).

## CD57 Expression during Infection

Chronic viral infections such as HCMV (104), human immunodeficiency virus (HIV) (105), hepatitis C virus (106), and Epstein–Barr virus (EBV) (107) infections offer some of the clearest examples of expansion of CD57^+^CD8^+^ T cells, presumably as a result of persistent antigenic stimulation, and increased proportions of CD57^+^CD8^+^ T cells have also been reported in those infected with human parvovirus (108), measles (109), pulmonary tuberculosis (92), and toxoplasmosis (93). The majority of these CD57^+^CD8^+^ T cells, at least in HCMV infection, appear to be antigen-specific and their presence is associated with a low incidence of reactivation (94, 95). Similar skewing of NK cells toward the CD57^+^ phenotype is now reported in a variety of viral infections (Table 2).

Increased frequencies of CD57^+^CD16^+^ NK cells were first reported in HCMV-infected individuals by Gratama et al. (110) and have been repeatedly confirmed (99, 111, 112). Studies of hematopoietic stem cell transplantation (HSCT) have been particularly informative, allowing detailed comparison of stem cell differentiation into NK cells in HCMV-infected and uninfected transplant recipients (111, 112) with rapid and persistent expansion of CD57^+^ NK cells that are also NKG2C^+^, KIR^+^, CD158b^+^, and potent producers of IFN-γ after stimulation with MHC Class I-deficient target cells, only in the HCMV-infected group (111). We now know that HCMV drives expansion of NKG2C^+^ NK cells and that these cells preferentially acquire CD57 (97–99, 111, 112). In HCMV-uninfected donors, there are roughly equal proportions of CD57^+^NKG2C^+^ and CD57^−^NKG2C^+^ NK cells whereas the ratio of CD57^+^NKG2C^+^ to CD57^−^NKG2C^+^ NK cells ranges from <1 to >60 in HCMV-infected donors (99); whether this variation reflects varying duration of HCMV infection is not known. HCMV reactivation after HSCT is associated with a threefold increase in the ratio of CD57^+^NKG2C^+^ to CD57^−^NKG2C^+^ NK cells within one year (111). Yet, in the absence of HCMV infection, NKG2C^+^ NK cells are no more likely to acquire CD57 than are NKG2C^−^ NK cells (112), suggesting that either binding of NKG2C to specific HCMV ligands or chronic viral infection *per se* drives NK cell differentiation. Importantly, CD57^+^CD16^+^ NK cells can kill HCMV-infected target cells (96) and this may be dependent upon, or enhanced by, α-HCMV antibodies (113).

While HCMV remains the clearest example of infection driving NK cell differentiation, other viral infections may cause a similar effect. For example, there is a three to fourfold expansion of the NK cell pool during acute hantavirus infection; NK cell numbers peak approximately 10 days after the onset of symptoms and remain above baseline for at least 60 days (114). This expansion is restricted to the NKG2C^+^ NK cell subset and the majority of these cells are CD57^+^, KIR^+^ and highly responsive to MHC Class I-deficient target cells. Hantavirus-infected endothelial cells express high levels of the NKG2C ligand HLA-E and expansion of the NKG2C^+^ NK cell subset is seen only in HCMV seropositive hantavirus patients, suggesting that hantavirus-induced HLA-E expression and/or inflammatory cytokines released during infection may drive the expansion and subsequent maturation of NKG2C^+^ NK cells that have been induced or “primed” by HCMV infection (114). Similarly, transient expansion of the CD57^+^ NKG2C^+^ NK cell population during acute chikungunya virus infection is also associated with HCMV seropositivity (115).

Expansion of the NKG2C^+^CD57^+^ NK cell subset has also been reported in HCMV^+^ individuals with chronic hepatitis B and hepatitis C infections, although the proportions of these cells did not differ markedly from previous reports in HCMV-infected but hepatitis virus-uninfected donors, leading the investigators to conclude that HCMV, rather than viral hepatitis, is the underlying driver of NK cell differentiation (97). In line with this, no association was found between expansion of the NKG2C^+^CD57^+^ NK cell subset and clinical indicators of hepatitis such as viral load or liver enzyme concentrations (97).

In HIV-infected individuals, the absolute number of CD57^+^ NK cells is stable and comparable to HIV-negative individuals but the ratio of CD57^+^ to CD57^−^ NK cells is higher than in uninfected individuals due to a gradual loss of CD57^−^ cells (which are highly dependent on monocyte and T cell-derived cytokines for their survival) (100). Unfortunately, the HCMV status of these subjects was not reported and may confound the comparison between the HIV^+^ and HIV^−^ individuals. Indeed, in another study, the positive association between frequency of NKG2C^+^ NK cells and HIV-1 infection disappears when adjusted for HCMV status (101). Nonetheless, it is also the case that the frequency of NKG2C^+^(CD57^+^) NK cells is higher in HCMV seropositive donors with HIV-1 infection than in HCMV seropositive donors without HIV-1 infection (102), suggesting either that – as for hantavirus or chikungunya virus – HIV-1 infection drives expansion of the HCMV-induced NKG2C^+^ population or that HIV-1 infected individuals experience more frequent reactivation of HCMV which then expands the NKG2C^+^ population. Significantly, CD57^+^ NK cells of HIV^+^ individuals retain a highly differentiated phenotype (CD16^+^KIR^+^perforin^+^) but have defects in degranulation (100) suggesting that they may have reduced cytotoxic potential. Finally, although no association was seen between accumulation of CD57^+^ NK cells and recurrence of genital herpes lesions due to herpes simplex virus 2 (HSV-2) infection (116), interpretation of this study is hindered by the lack of an HSV-2-uninfected control group.

There have been very few studies of NK cell subsets in the context of bacterial or parasitic infections. Patients with chronic Lyme Disease (*Borrelia burgdorferi*) have lower proportions of peripheral blood CD57^+^ NK cells compared to those with acute disease and uninfected controls and this phenotype was maintained for over 10 years in one person with persistent infection (117, 118). In contrast, no significant differences in numbers of peripheral blood CD3^−^CD57^+^ cells were noted between patients with post-Lyme disease syndrome, individuals recovered from Lyme disease and healthy controls (119). The suggestion (118) that high frequencies of CD57^+^ NK cells may be a biomarker of Lyme disease progression thus seems premature, especially given the potential impact on NK cell phenotype of HCMV and other infections.

In summary, viral infections are important drivers of NK cell differentiation with HCMV playing a primary role in selecting for NKG2C^+^CD57^+^ cells and other viruses driving their expansion and differentiation.

## CD57 Expression and Aging

Given the enormous impact of infection on NK cell maturation and differentiation, it is not surprising that NK cell populations change with age, which is a proxy for cumulative exposure to infection and other physiological insults. At birth virtually no T cells express CD57 (120) but the proportion rises with age, reaching 20–30% in young adults (20); by 80 years of age 50–60% of CD8^+^ T cells are CD28^−^ (and thus likely CD57^+^) (20, 121). Similarly, with increasing age, increasing numbers of circulating NK cells are achieved by an expansion of the CD56^dim^ and CD57^+^ subsets and an absolute, as well as a proportional, decline in CD56^bright^ cells (35, 53–55, 122–125). At birth, all CD56^dim^ NK cells are CD57^−^; among European adults (18–60 years of age) 25–60% of CD56^dim^ NK cells are CD57^+^ and this continues to increase slightly, but significantly, after the age of 80 years (124). Interestingly, CD56^dim^CD57^+^ NK cells accumulate very rapidly in an African (Gambian) population reaching adult levels (20–70%) by the age of 5 years (Goodier et al. unpublished); this may reflect very high HCMV seroprevalence rates in this age group in this community.

The increased proportion of CD56^dim^CD57^+^ NK cells in the elderly likely explains the maintenance of NK cell cytotoxic responses despite reduced responsiveness to cytokine stimulation [reviewed in Ref. (56)], however, the significance of these changes in terms of overall immune competence is poorly understood. The gradual loss of the CD56^bright^ NK cell population, and the consequent decline in NK-derived cytokines that activate dendritic cells and monocytes, has been assumed to contribute to age-associated declines in immune competence but the potential counterbalancing effect of an increased proportion of highly cytotoxic CD57^+^ NK cells has received little attention (123). Comprehensive studies are now needed to assess the cytokine-producing and cytotoxic function of individual NK cell subsets in response to cytokine stimulation as well as activation via CD16 and NCRs and the extent to which this changes with age and HCMV status.

## Conclusion and Future Directions

CD57 is a very useful marker of NK cell maturation, identifying cells with potent cytotoxic potential but decreased sensitivity to cytokines and reduced replicative potential. CD57^+^ NK cells appear to be a stable sub-population, increasing with age and exposure to pathogens (especially, but not exclusively, HCMV) and their presence is consistently associated with better outcomes in cancer and autoimmune disease. However, the majority of clinical studies have been cross-sectional, with limited follow up and data on crucial confounding factors such as HCMV infection are typically lacking. Recent studies of HSCT (111, 112) demonstrate the power of prospective and longer term studies in beginning to assign causality in terms of NK cell phenotype, function, and disease. Nevertheless, precise understanding of the role of CD57 expression on NK cells requires a detailed dissection of the underlying biology of CD57, about which very little is known. Given that there is no evidence that CD57 is expressed on murine NK cells, this is not a simple task. Possible approaches in human NK cells might include conducting a comprehensive analysis of NK cell molecules expressing CD57, blocking CD57 in *in vitro* functional NK cell assays, or manipulating expression or enzymatic activity of B3GAT1 (the key enzyme in the biosynthesis of CD57) using RNA interference or specific inhibitors.

## Conflict of Interest Statement

The authors declare that the research was conducted in the absence of any commercial or financial relationships that could be construed as a potential conflict of interest.
